# Supplementary material for: The autophagy protein ATG9A enables lipid mobilization from lipid droplets
Source: Nat Commun. 2021 Nov 19;12:6750. doi: 10.1038/s41467-021-26999-x (PMC8605025; doi:10.1038/s41467-021-26999-x)
Supplement: Supplementary file 1 — Supplementary Information [file 41467_2021_26999_MOESM1_ESM.pdf]

Supplementary Information

The autophagy protein ATG9A enables lipid mobilization from lipid droplets

Elodie Mailler[1], Carlos M. Guardia[1], Xiaofei Bai[2], Michal Jarnik[1], Chad D. Williamson[1], Yan Li[3], Nunziata Maio[4], Andy Golden[2] and Juan S. Bonifacino[1*]

[1] Neurosciences and Cellular and Structural Biology Division, *Eunice Kennedy Shriver* National Institute of Child Health and Human Development, National Institutes of Health, Bethesda, Maryland, USA.

[2] Laboratory of Biochemistry and Genetics, National Institute of Diabetes and Digestive and Kidney Diseases, National Institutes of Health, Bethesda, Maryland, USA.

[3] Proteomics Core Facility, National Institute of Neurological Disorders and Stroke, National Institutes of Health, Bethesda, Maryland, USA.

[4] Metals Biology and Molecular Medicine Group, *Eunice Kennedy Shriver* National Institute of Child Health and Human Development, Bethesda, MD, USA

*Corresponding author: juan.bonifacino@nih.gov

Short title: ATG9A enables lipid mobilization from lipid droplets

Supplementary fig. 1

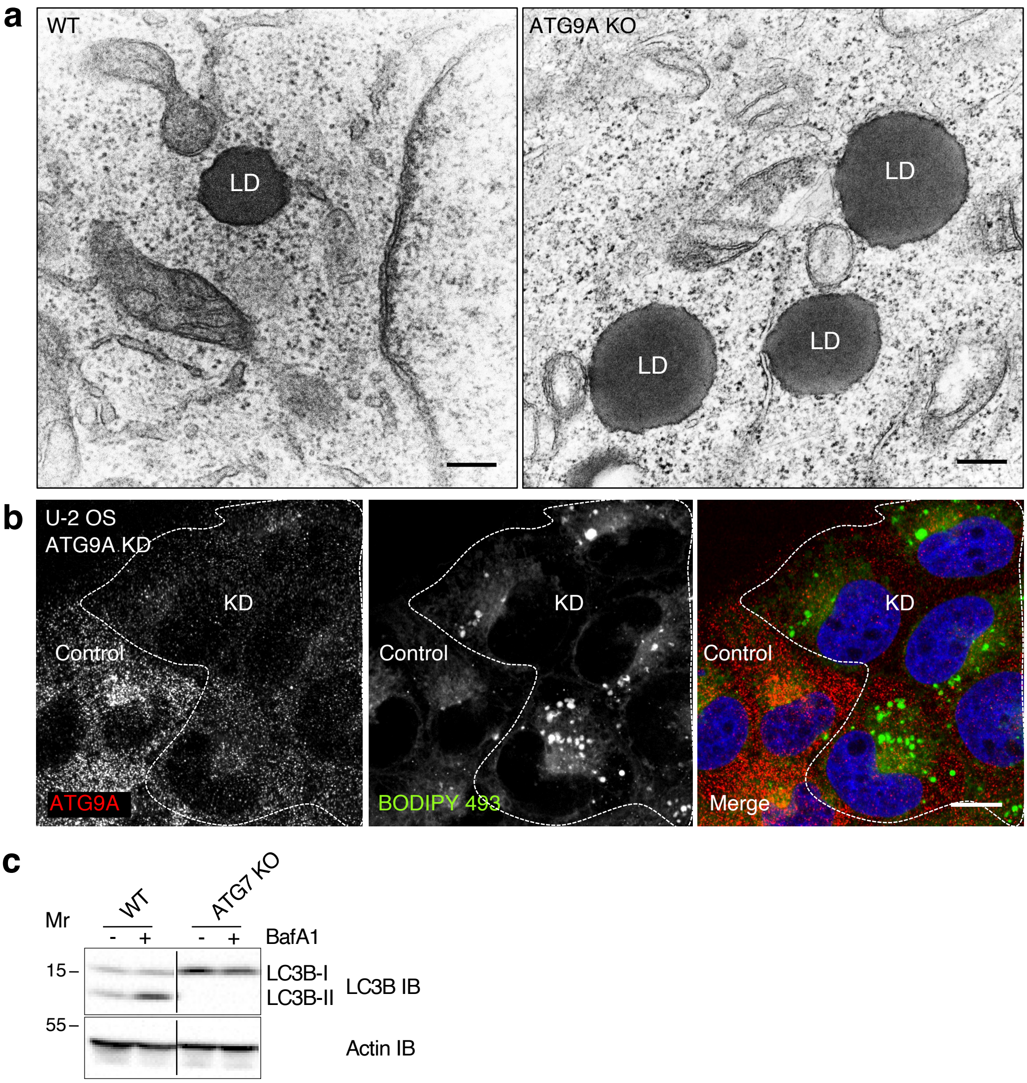

Supplementary fig. 1: Ultrastructure and phenotypic rescue of LDs in ATG9A-depleted cells.

a, TEM of WT and ATG9A-KO HeLa cells. Scale bars: 200 nm. Notice the increased number and size, but similar round shape, of LDs in ATG9A-KO cells compared to WT cells (three independent experiments). **b**, Confocal fluorescence microscopy of U-2 OS cells treated with ATG9A siRNA (ATG9A KD), fixed, permeabilized and stained for LDs with BODIPY 493 (green), with an antibody to ATG9A (red) and for nuclei with DAPI (blue). Single-channel images are shown in grayscale. Groups of control and KD cells are delineated. Scale bar: 10 μm. Notice the increased number and size of LDs in KD cells (three independent experiments). **c**, SDS-PAGE and IB, using antibodies to LC3B and actin, of WT and ATG7-KO HeLa cells incubated for 4 h with 100 nM bafilomycin A1 (BafA1). The positions of molecular mass (Mr) markers (in kDa) are indicated on the left. Results are representative from three independent experiments with similar results.

Supplementary fig. 2

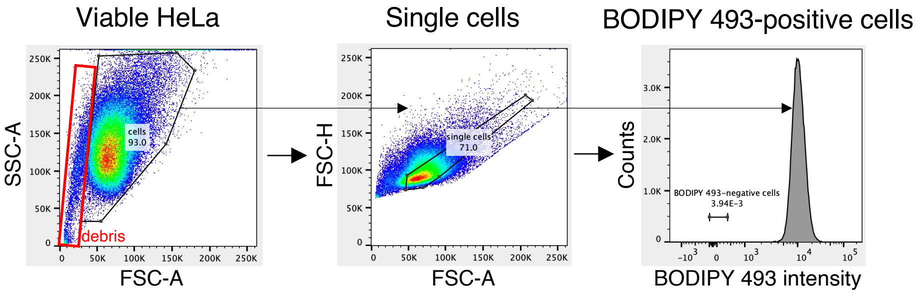

Supplementary fig. 2: Gating strategy for the measurement of neutral lipids in WT and KO HeLa cells by FACS analysis.

Gating strategy used to measure neutral lipid levels in HeLa cells by BODIPY 493 staining and FACS analysis (Fig. 2 a,b). HeLa cells were incubated for 2 h at 37°C with or without 200 μM oleate (OA dissolved in methanol), and stained (+) or not (-) with BODIPY 493. Viable cells were first gated on a plot of SSC-A vs. FSC-A. Single cells were next gated on a plot of FSC-H *vs.* FSC-A, and a gate for BODIPY 493-unlabeled HeLa cells was set. All flow cytometry analyses were performed on 100,000 cells. The data panels presented here correspond to one of the three biological replicates of WT HeLa treated with methanol and stained with BODIPY 493.

Supplementary fig. 3

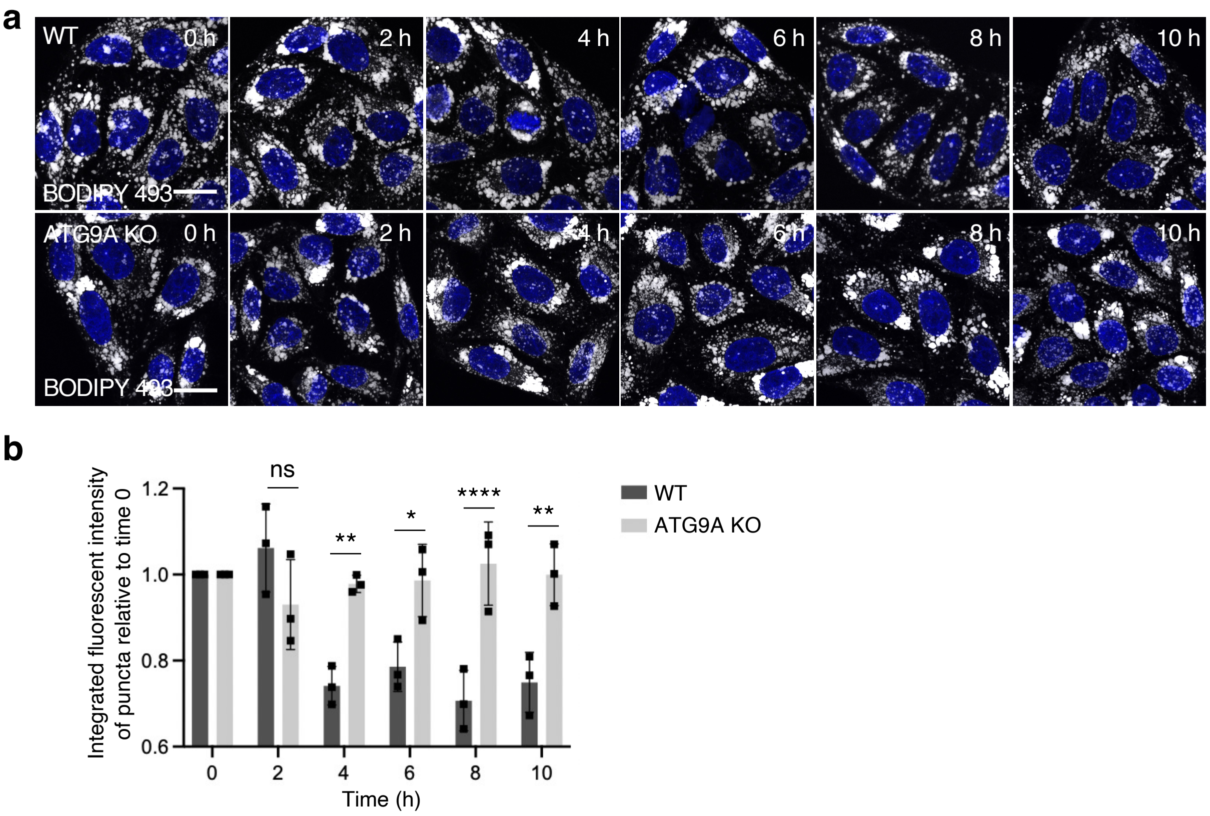

Supplementary fig. 3: Time course of LD decrease upon OA removal from the medium.

a, WT and ATG9A-KO cells were incubated for 16 h at 37°C with 1 μM OA, washed, cultured in complete medium (CM), fixed at the indicated times, permeabilized, stained for LDs with BODIPY 493 (green) and for nuclei with DAPI (blue), and imaged by confocal fluorescence microscopy. BODIPY 493 staining is shown in grayscale and DAPI staining in blue. Scale bars: 10 μm. Results are representative from three independent experiments with similar results. **b**, Quantification of LD intensity per cell using the 'Analyze particles' function of ImageJ in 25-35 cells in each of three independent experiments. Bar graphs represent the mean ± SD fold-change of these values relative to WT or ATG9A-KO cells at time 0. Statistical significance was determined using two-sided ANOVA with Tukey post-hoc test (ns $p>0.05$, * $p<0.05$, ** $p<0.01$, **** $p<0.0001$).

Supplementary fig. 4

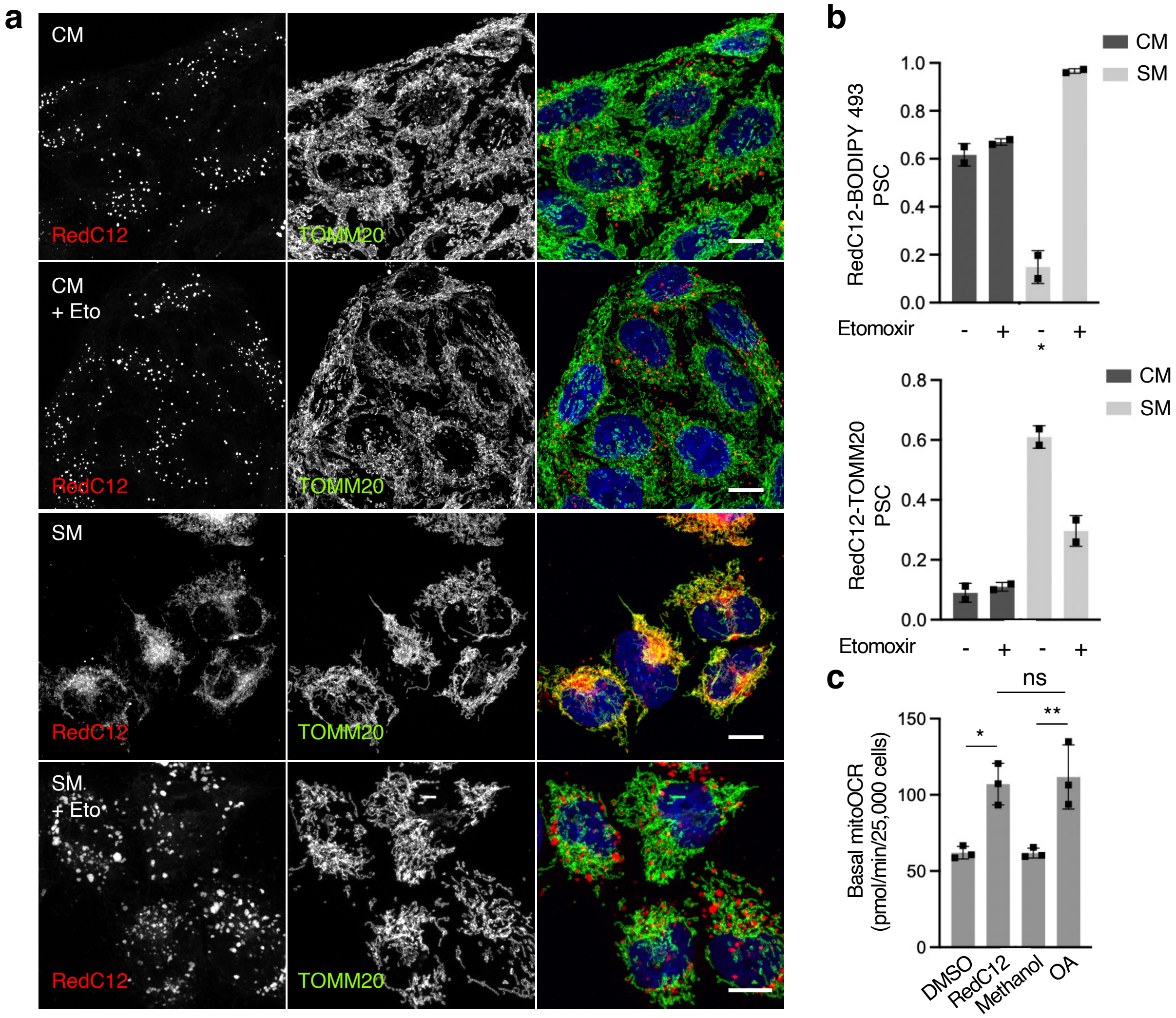

Supplementary fig. 4: Effect of etomoxir and OA on FA trafficking and mitochondrial metabolism in WT cells.

a, WT HeLa cells were pulsed for 16 h with RedC12 (red), chased for 24 h in complete medium (CM) or amino-acid- and serum-free medium (SM) with 4 μM etomoxir, fixed, stained mitochondria with antibody to TOMM20 (green), and nuclei with DAPI (blue), and imaged by confocal fluorescence microscopy. Single-channel images are shown in grayscale. Scale bars: 10 μm. Notice that etomoxir blocks the transfer of RedC12 to mitochondria in SM. **b**, Quantification of co-localization of RedC12 with LDs (stained with BODIPY 493) and mitochondria. The PSC between signals in the two channels was calculated by using the PSC colocalization plugin in ImageJ. Bar graphs represent the mean ± SD from 25-35 cells per biological replicate in two independent experiments. **c**, Quantification of basal mitochondrial OCR in WT cells supplied with two different sources of FAs, OA and RedC12. Mitochondrial OCR was calculated by subtracting the non-mitochondrial OCR from the basal OCR in the absence or presence of the indicated additives. Bar graph represents the mean ± SD of OCR values from three independent experiments. Statistical significance was determined using one-sided ANOVA with Tukey post hoc test (ns $p>0.05$, * $p<0.05$, ** $p<0.01$).

Supplementary fig. 5

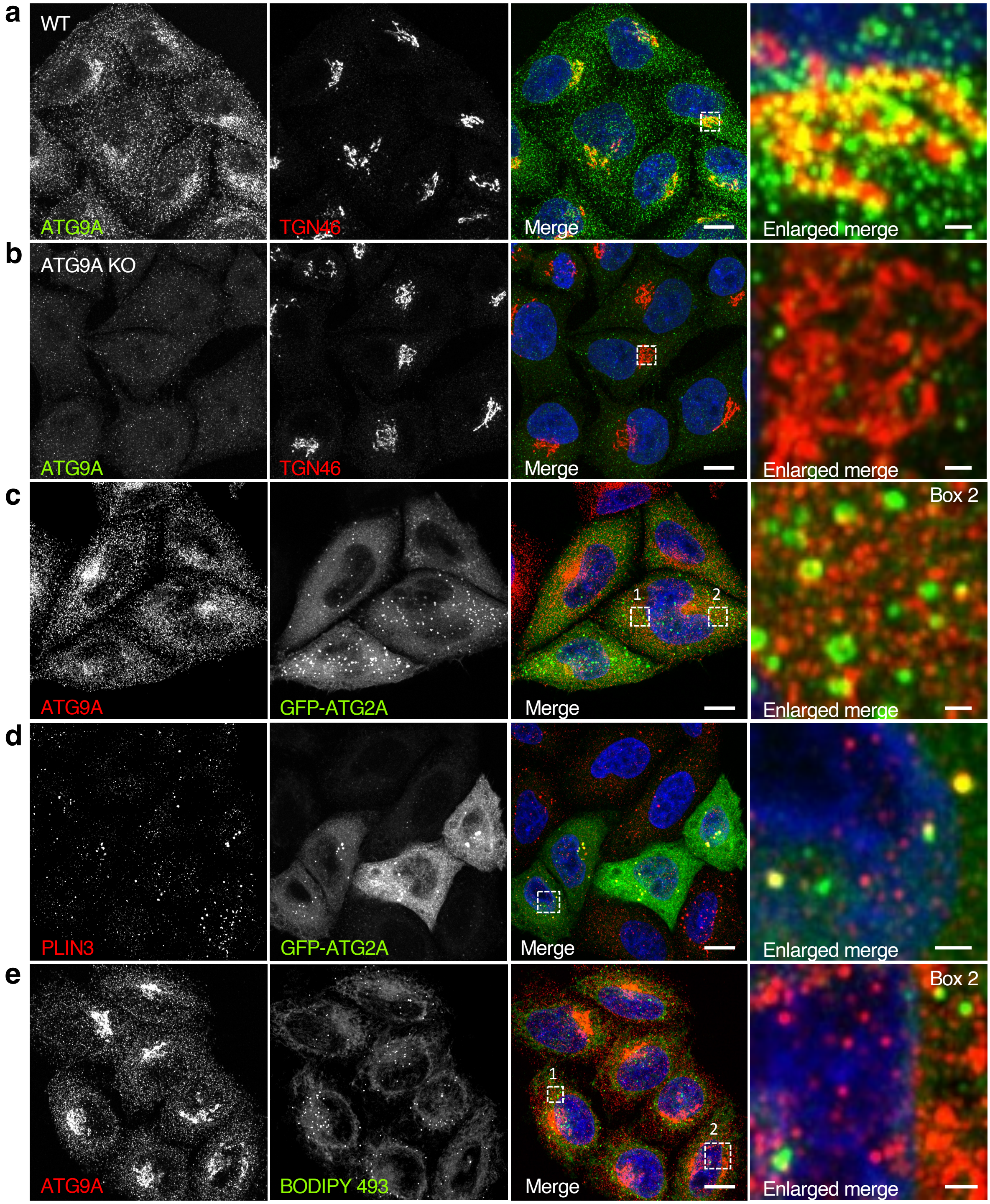

Supplementary fig. 5: Immunofluorescence microscopy of ATG9A relative to other organellar markers.

a, Confocal immunofluorescence microscopy of WT HeLa cells that were fixed, permeabilized and stained with antibodies to endogenous ATG9A (green) and TGN46 (red). Nuclei were stained with DAPI (blue). **b**, Same as panel **a** but using ATG9A-KO HeLa cells. **c**, Confocal immunofluorescence microscopy of WT HeLa cells transfected with a plasmid encoding GFP-ATG2A (green), fixed, permeabilized and stained with an antibody to endogenous ATG9A (red) and with DAPI (blue). **d**, Same as panel **c**, but WT cells were stained with an antibody to endogenous PLIN3 (red) instead of ATG9A. **e**, Same as panel **a**, but WT cells were stained with an antibody to endogenous ATG9A (red) and with BODIPY 493 (green) instead of an antibody to TGN46. Scale bars: 10 μm. Enlarged views of boxes labeled 1 are shown in panels **a** and **c** of Fig. 6. Enlarged views of unlabeled boxes or boxes labeled 2 are shown on the right column. Scale bar: 1 μm. Notice the co-localization of perinuclear ATG9A with TGN46 (**a**), the specificity of staining for ATG9A (**b**), the largely different localization of ATG9A relative to LDs stained for GFP-ATG2A (**c**) and BODIPY 493 (**e**), and the localization of GFP-ATG2A to LDs stained for PLIN3 (**d**). **a-e**, Results are representative from three independent experiments with similar results.

Supplementary fig. 6

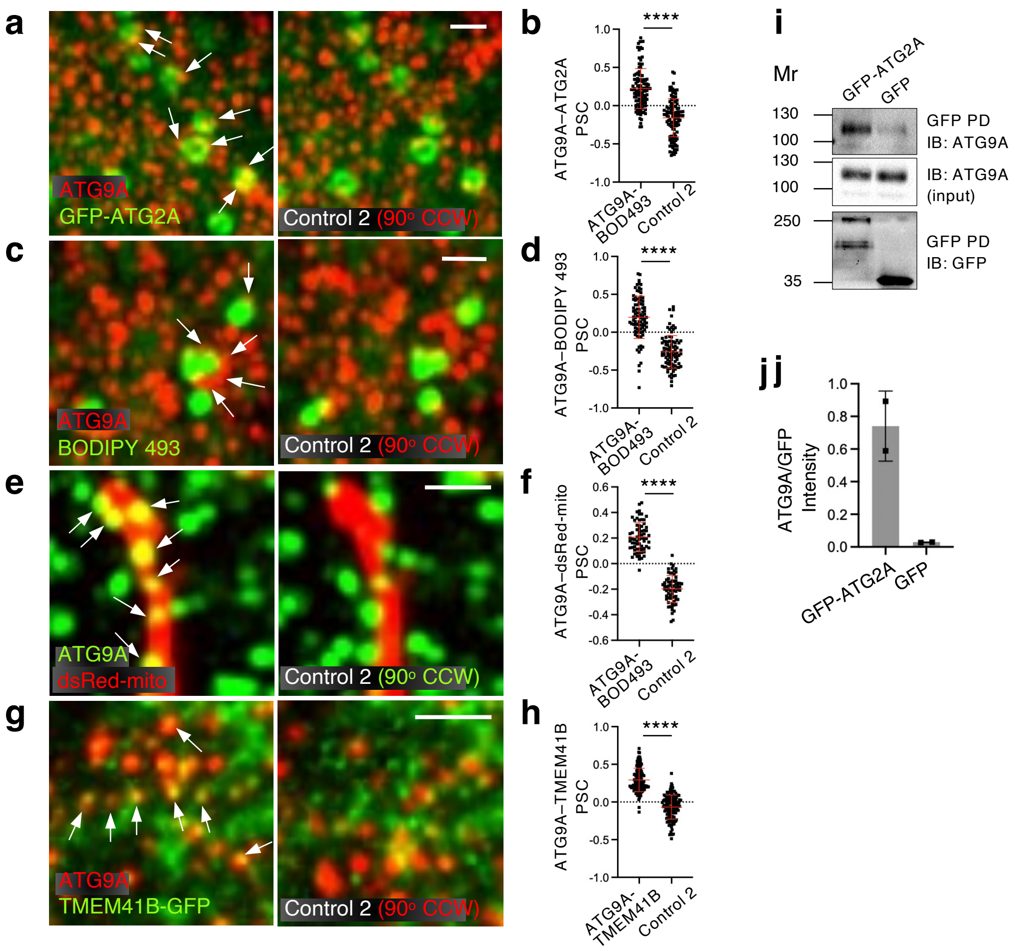

Supplementary fig. 6: Additional controls of ATG9A co-localization with other proteins, and pulldown of ATG9A with ATG2A.

a-h, Same analysis as in Fig. 6a-h, except that merge images were compared to an alternative random co-localization control in which the red channel was rotated 90° counter-clockwise (CCW). Scale bars: 1 μm (three independent experiments). **i**, Analysis of ATG2A-ATG9A interaction by GFP pulldown (PD) and immunoblotting (IB). WT HeLa cells were transiently transfected with plasmids encoding GFP-ATG2A or GFP (control). Cell extracts were incubated with anti-GFP beads, and bound and input proteins were analyzed by SDS-PAGE and immunoblotting with antibodies to ATG9A and GFP. The positions of molecular mass (Mr) markers (in kDa) are indicated on the left. **j**, Quantification of the ratio of ATG9A in the PD relative to GFP in the input in the experiment shown in **i**. Bars represent the mean ± SD from two independent experiments.

Supplementary fig. 7

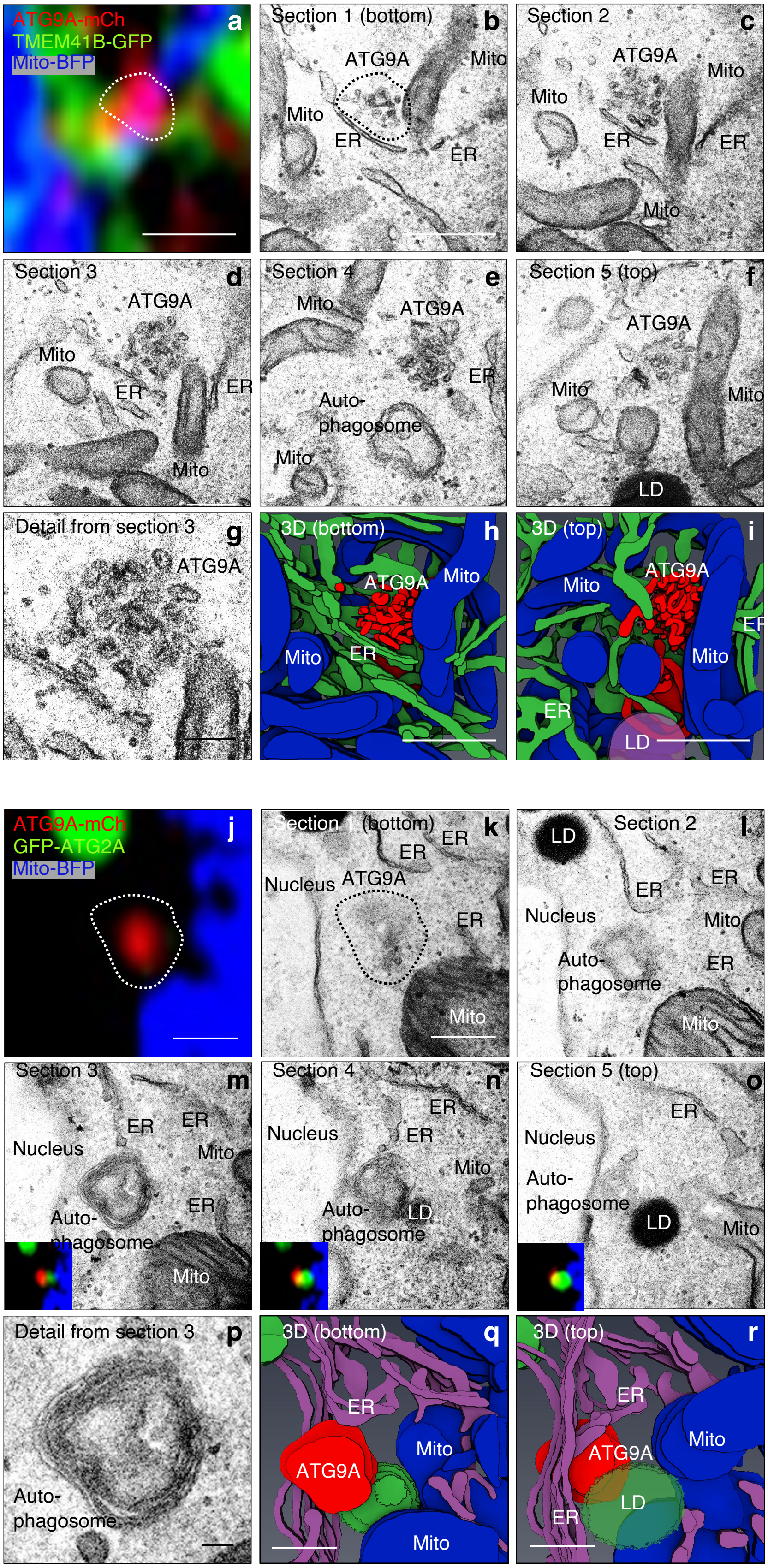

Supplementary fig. 7: Two additional examples of CLEM of ATG9A structures relative to other organelles.

Analysis was done as described for Fig. 8. HeLa cells were transiently transfected with plasmids encoding ATG9A-mCherry (red), mito-BFP (blue) and TMEM41B-GFP (green) in **a-i** or GFP-ATG2A in **j-r**. Cells were starved for 20 min prior to fixation and analysis by CLEM. **a**,**j**, Airyscan image of an ATG9A-positive structure (outlined) near TMEM41B (**a**) or ATG2A (**j**) structures and mitochondria. Scale bar: 0.5 μm. **b**,**k**, Transmission EM (TEM) image corresponding to the Airyscan image shown in panel **a**,**j**. Scale bar: 0.5 μm. **c**-**f**, **l-o**, TEM images of sequential serial sections. **g**,**p**, Enlarged view of the ATG9A-positive structure in **b**,**k**. Scale bar: 0.1 μm. **h**,**i**,**q**,**r** 3D reconstruction of TEM images in panels **b**-**f**, **k-o** using Amira software, viewed from the bottom (**h**,**q**) or the top (**i**,**r)** of the cell. The identity of different organelles is indicated. Scale bar: 0.5 μm Results are representative from two independent experiments with similar results.

Supplementary fig. 8

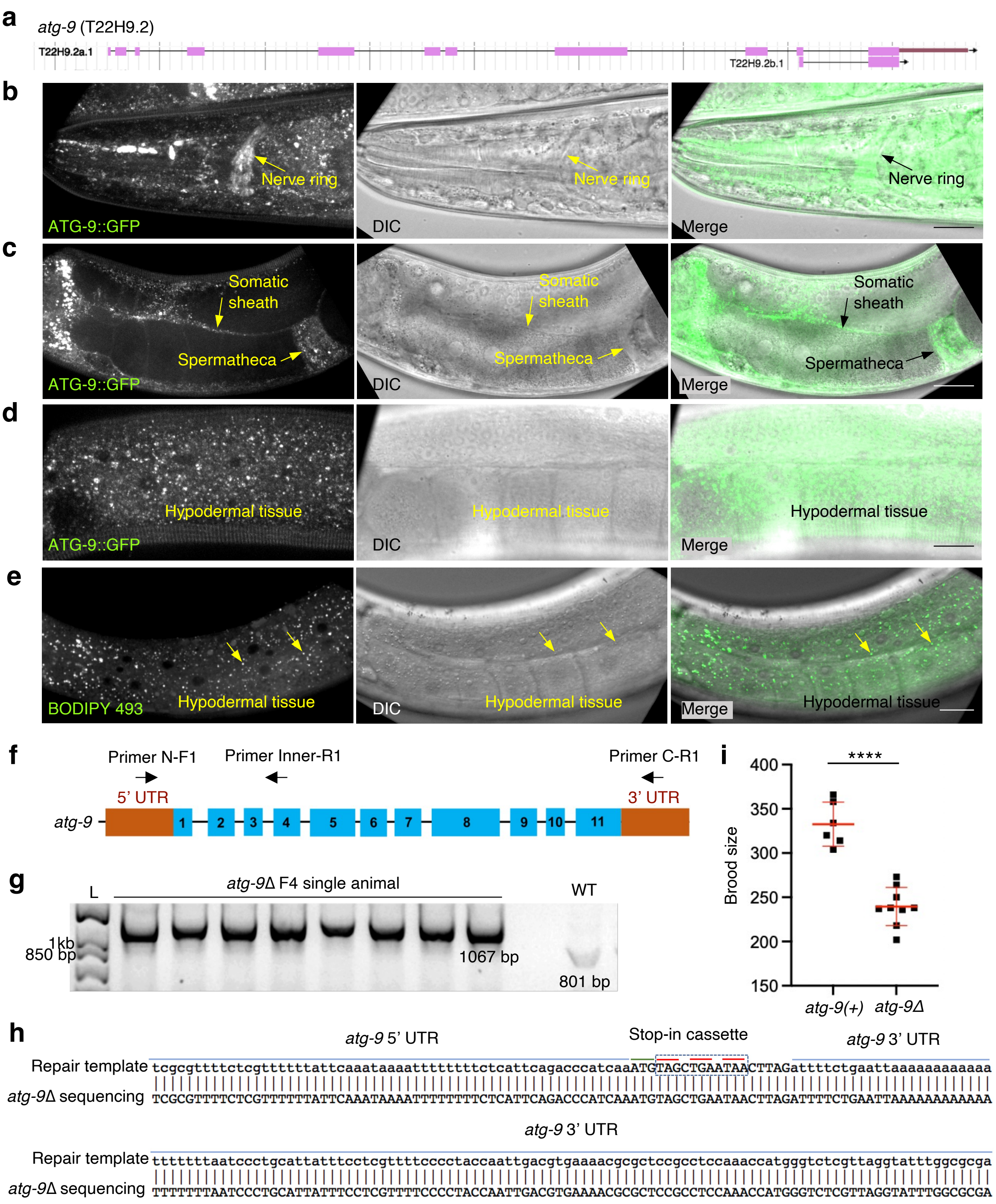

Supplementary fig. 8: Expression pattern of ATG-9::GFP in *C. elegans* and verification of deletions in *atg-9* knockout animals.

a, *C. elegans atg-9* encodes two mRNA isoforms, T22H9.2a.1 and T22H9.2b.1, according to WormBase (https://wormbase.org//#012-34-5). T22H9.2b.1 encodes only the last two exons of *atg-9*. The 5′-3′ orientation is left to right. **b-d**, Fluorescence and DIC microscopy of WT *C. elegans* showing expression of endogenously-tagged ATG-9::GFP in multiple tissues, including the nerve ring, somatic sheath, spermatheca and hypodermis. Left panels show ATG-9::GFP, middle panels DIC and right panels merged images. Scale bars: 20 μm. Results are representative from three independent experiments with similar results. **e**, Fluorescence and DIC images of hypodermal tissue from a WT animal stained with BODIPY 493. Scale bar: 20 μm (three independent experiments). **f**, Genomic structure of *atg-9* indicating the position of exons and genotyping primers used to verify the KO allele. **g**, Representative PCR gel from genotyping single animals for *atg-9Δ* candidates. The flanking primers N-F1 and C-R1, located outside the deleted region, and an internal primer Inner-R1, were used to genotype for homozygosity of candidate *atg-9(av244Δ)* full-deletion animals. A homozygous deletion was identified by a 1,067-bp band amplified with N-F1 and C-R1. WT animals were identified by an 801-bp band amplified by N-F1 and Inner-R1 (three independent experiments). **h**, Homozygous *atg-9Δ* animals were confirmed by Sanger sequencing, including the identification of a "stop-in cassette" (red lines) after the start codon. **i**, Brood size was significantly reduced in *atg-9Δ* when compared with WT animals. Data are presented as mean ± SD. Statistical significance was calculated using an unpaired two-tailed Student's t-test (**** $p<0.0001$).

Supplementary table 1: list of primers used in this study.

Name of primers	Purpose	Sequence of primers (5′ to 3′)
SYP1, SYP2, SYP3 and SYP4	Guide RNAs sequences used to generate ATG9A-KO HeLa	5-CACCGTATAGGAGGCCTCTAGGCGC<br>5′-AAACGCGCCTAGAGGCCTCCTATAC<br>5′-CACCGCTGTTGGTGCACGTCGCCGA<br>5′-AAACTCGGCGACGTGCACCAACAGC
EM216 and EM217	Guide RNA sequence used to generate ATG2A-KO HeLa	5′-CACCGGCTCAGCCTCGATCTGTACA<br>5′-AAACTGTACAGATCGAGGCTGAGCC
EM222 and EM223	Guide RNA sequence used to generate ATG2B-KO HeLa	5′-CACCGCCTCTGCAGGAGGTACCGGC<br>5′-AAACGCCGGTACCTCCTGCAGAGGC
EM47 and EM48	Guide RNA sequence used to generate ATG7-KO HeLa	5′-CACCGAACTGCAGTTTAGAGAGTCC<br>5′-CGGACTCTCTAAACTGCAGTTCAAA
SYP1 and SYP2	Primers used to generate ATG9A-GFP construct in pEGFP-N1	5′-AGCTCAAGCTTCGAATTCTGATGGCGCAG TTTGACACTGA<br>5′-GCCCGCGGTACCGTCGACGTTACCTTGTG CACCTGAGGGG
CG1 and CG2	Primers used to generate ATG9A-GFP 1-723 construct in pEGFP-N1	5′-CAGCTCCACAAGCAGCAGGCCCAGGCTAC GTCGACGGTACCGCGGGCCCGGGAT<br>5′-ATCCCGGGCCCGCGGTACCGTCGACGTAGC CTGGGCCTGCTGCTTGTGGAGCTG
CG3 and CG4	Primers used to generate ATG9A-GFP 1-522 construct in pEGFP-N1	5′-GGTGTGGGAGATACCTGCTCCTTTGCTACGT CGACGGTACCGCGGGCCCGGGAT<br>5′-ATCCCGGGCCCGCGGTACCGTCGACGTAGC AAAGGAGCAGGTATCTCCCACACC
EM153 and EM154	Primers used to generate ATG9B-GFP construct in pEGFP-N1	5′-GTTTAAACGGGCCCTCTAGAATGGTGAGCC GAATGGGCTG<br>5′-CGAATCCGGATCCGGTACCGTCAGTGCAAG AGGCCCGGT
EM214 and EM215	Primers used to generate TMEM41B-GFP in pEGFP-N1	5-GTGGACAGGGGGCTGTGGTGATGGCGAAAG GCAGAGTCGC<br>5′-ACTGCAGAATTCGAAGCTTCTCAAATTTCTG CTTTAGTT

Name of primers	Purpose	Sequence of primers (5′ to 3′)
CG5 and CG6	Primers used to generate GFP-RUSC2 in pEGFP-C1	5′-GAATTCTGCAGTCGACGGTACCATGGATAG TCCCCCAAAGCTGAC<br>5′-TCAGTTATCTAGATCCGGTGGATTCAGTTTT GGCTGCTTCCAGGGG
EM212 and EM213	Primers used to generate TMEM41B-FTS in pcDNA3.1	5′-GTTTAAACGGGCCCTCTAGAATGGCGAAAG GCAGAGTCGC<br>5′-TCGAATCCGGATCCGGTACCCTCAAATTTCT GCTTTAGTT
EM344 and EM345	Primers used to generate CD63-FTS in pcDNA3.1	5′-GTTTAAACGGGCCCTCTAGAATGGCGGTGG AAGGAGGAAT<br>5′-TCGAATCCGGATCCGGTACCCATCACCTCG TAGCCACTTC
XB1 and XB2	Guide RNAs sequences used to generate atg9Δ worms	N-terminus guide RNA:<br>5′- ACTGTGAGTTGAACATTTGA<br>C-terminus guide RNA:<br>5′-ATCTAAGAGCTAAAGCTAAG
XB3	Repair template to generate atg9Δ worms	5′-aaaaaattttttttttaattcagaaaatCTAAGTTATTCAG CTACATttgatgggtctgaatgagaaaaaaaattttatttgaat
XB4, XB5 and XB6	Primers used to genotype WT and atg9Δ worms	F1: 5′-TTCTAACAGTCACCACCCGC<br>R1: 5′-GTCAACAACGGGCTGCTTTT<br>Inner: 5′-TTTTCGGGGGTTTCCTGGAC

Supplementary table 2: *C. elegans* strains list in the study.

	Strain	Genotype
Fig. 9	N2	Bristol (wild-type)
	AG608	atg-9(av244Δ)V. CRISPR-Cas9 Edit, deletion of coding region.
	AG612	atg-9(ola274[atg-9::gfp]) V; seip-1(av169[seip-1::mScarlet]) V.
Supplementary fig. 8	N2	Bristol (wild-type)
	AG608	atg-9(av244Δ)V. CRISPR-Cas9 Edit, deletion of coding region.
	DCR4521	atg-9(ola274[atg-9::gfp]) V
