# Supplementary material for: The autophagy protein ATG9A enables lipid mobilization from lipid droplets
Source: Nat Commun. 2021 Nov 19;12:6750. doi: 10.1038/s41467-021-26999-x (PMC8605025; doi:10.1038/s41467-021-26999-x)
Supplement: Supplementary file 3 — Description of Additional Supplementary Files [file 41467_2021_26999_MOESM3_ESM.pdf]

Description of Additional Supplementary Files

File name: Supplementary Data 1

Description: Raw data of the TAP-MS analysis of proteins that co-purify with ATG9A-FTS in HeLa cells.
