# Supplementary material for: The autophagy protein ATG9A enables lipid mobilization from lipid droplets
Source: Nat Commun. 2021 Nov 19;12:6750. doi: 10.1038/s41467-021-26999-x (PMC8605025; doi:10.1038/s41467-021-26999-x)
Supplement: Supplementary file 5 — Reporting Summary [file 41467_2021_26999_MOESM5_ESM.pdf]

nature research

Corresponding author(s): Juan S. Bonifacino

Last updated by author(s): Oct 5, 2021

Reporting Summary

Nature Research wishes to improve the reproducibility of the work that we publish. This form provides structure for consistency and transparency in reporting. For further information on Nature Research policies, see our Editorial Policies and the Editorial Policy Checklist.

Statistics

For all statistical analyses, confirm that the following items are present in the figure legend, table legend, main text, or Methods section.

n/a	Confirmed	
[ ]	[x]	The exact sample size (n) for each experimental group/condition, given as a discrete number and unit of measurement
[ ]	[x]	A statement on whether measurements were taken from distinct samples or whether the same sample was measured repeatedly
[ ]	[x]	The statistical test(s) used AND whether they are one- or two-sided<br>*Only common tests should be described solely by name; describe more complex techniques in the Methods section.*
[x]	[ ]	A description of all covariates tested
[x]	[ ]	A description of any assumptions or corrections, such as tests of normality and adjustment for multiple comparisons
[ ]	[x]	A full description of the statistical parameters including central tendency (e.g. means) or other basic estimates (e.g. regression coefficient) AND variation (e.g. standard deviation) or associated estimates of uncertainty (e.g. confidence intervals)
[ ]	[x]	For null hypothesis testing, the test statistic (e.g. F, t, r) with confidence intervals, effect sizes, degrees of freedom and P value noted<br>*Give P values as exact values whenever suitable.*
[x]	[ ]	For Bayesian analysis, information on the choice of priors and Markov chain Monte Carlo settings
[x]	[ ]	For hierarchical and complex designs, identification of the appropriate level for tests and full reporting of outcomes
[ ]	[x]	Estimates of effect sizes (e.g. Cohen's d, Pearson's r), indicating how they were calculated

Our web collection on statistics for biologists contains articles on many of the points above.

Software and code

Policy information about availability of computer code

Data collection	Mass spectrometry data: Proteome Discoverer 2.4
Data analysis	Flow cytometry: FlowJo version 10 (BD, Ashland, OR)<br>Microscopy: open-source software Fiji ImageJ version 2.0.0 (NIH, Bethesda, MD), Zen Black software version 14.0.18.201 (Zeiss, Oberkochen, Germany)<br>Statistics: commercially available software GraphPad Prism version 8.3.1 (GraphPad software, San Diego, CA)<br>CLEM 3D reconstruction: commercially available software Amira version 6.5.0 (ThermoScientific, Waltham, MA)<br>Filtering of mass spectrometry data: CRAPome database at www.crapome.com

For manuscripts utilizing custom algorithms or software that are central to the research but not yet described in published literature, software must be made available to editors and reviewers. We strongly encourage code deposition in a community repository (e.g. GitHub). See the Nature Research guidelines for submitting code & software for further information.

Data

Policy information about availability of data

All manuscripts must include a data availability statement. This statement should provide the following information, where applicable:

- Accession codes, unique identifiers, or web links for publicly available datasets
- A list of figures that have associated raw data
- A description of any restrictions on data availability

Source data for Figures 1b, 1d, 1e, 2b, 2d to 2h, 2j, 2k, 3b, 3c, 3e, 3f, 4c, 4e, 5e, 6b, 6d, 6f, 6h, 7c, 7d, 9d, 9e, supplementary figure 1c, supplementary figure 3b, supplementary figure 4b and 4c, supplementary figure 6b, 6d, 6f, 6h, 6i and 6j, supplementary figure 8g, 8i. Supplementary data 1 will be provided with this paper.

Field-specific reporting

Please select the one below that is the best fit for your research. If you are not sure, read the appropriate sections before making your selection.

- [x] Life sciences
- [ ] Behavioural & social sciences
- [ ] Ecological, evolutionary & environmental sciences

For a reference copy of the document with all sections, see nature.com/documents/nr-reporting-summary-flat.pdf

Life sciences study design

All studies must disclose on these points even when the disclosure is negative.

Sample size	No calculations were performed to predetermine sample size. The sample size was determined based on similar experiments previously conducted by our group and others on similar fields. We quantified at least 20 cells randomly selected per condition from at least three independent replicates in microscopy experiments, and immunoblots, immunoprecipitation, and seahorse assays were from at least three independent replicates. These particular sample sizes seem sufficient for our experiments since we usually detect differences of phenotypes between replicates with p-values lower than 0.01 and results are reproducible enough with this number of replicates.
Data exclusions	Transfected cells expressing a high level of proteins were explicitly excluded from analysis (pre-established criteria) since overexpression artifacts potentially affect cell morphology and exhibit saturated signal intensities.
Replication	Experiments were conducted multiple times and were reproducible, as indicated in the legends to each figure.
Randomization	Randomization was not done. We analyzed all samples for one experiment in the same way.
Blinding	Blinding was not done as cells were prepared and analyzed by the same investigator due to the sophistication required in each experiment.

Reporting for specific materials, systems and methods

We require information from authors about some types of materials, experimental systems and methods used in many studies. Here, indicate whether each material, system or method listed is relevant to your study. If you are not sure if a list item applies to your research, read the appropriate section before selecting a response.

Materials & experimental systems

n/a	Involved in the study	
[ ]	[x]	Antibodies
[ ]	[x]	Eukaryotic cell lines
[x]	[ ]	Palaeontology and archaeology
[ ]	[x]	Animals and other organisms
[x]	[ ]	Human research participants
[x]	[ ]	Clinical data
[x]	[ ]	Dual use research of concern

Methods

n/a	Involved in the study	
[x]	[ ]	ChIP-seq
[ ]	[x]	Flow cytometry
[x]	[ ]	MRI-based neuroimaging

Antibodies

Antibodies used	We used primary antibodies to the following proteins:<br>ATG2A (rabbit polyclonal, Proteintech, 23226-1-AP, 1/500), ATG2B (rabbit polyclonal, Proteintech, 25155-1-AP, 1/500), ATG7 (rabbit monoclonal, Cell Signaling, 8558, 1/1000), ATG9A (rabbit monoclonal, Abcam, 108338, IB: 1/2000, IF: 1/250), LC3B (rabbit monoclonal, Cell Signaling, 3868, 1/1000), PLIN3 (mouse polyclonal, Proteintech, 10694-1-AP, 1/500), PLIN5 (guinea pig polyclonal, PROGEN, GP31, 1/500), TGN46 (sheep polyclonal, Bio-Rad, AHP500G, , IF:1/500), TOMM20 (mouse monoclonal, Abcam, 56783, IF: 1/500).<br>We used the following secondary antibodies:<br>Alexa Fluor 488-conjugated donkey anti-rabbit IgG (Invitrogen, A21206, 1/1000), Alexa Fluor 488-conjugated donkey anti-mouse IgG (Invitrogen, A21202, 1/1000), Alexa Fluor 555-conjugated donkey anti-rabbit IgG (Invitrogen, A31572, 1/1000), Alexa Fluor 555-conjugated donkey anti-mouse IgG (Invitrogen, A31570, 1/1000), Alexa Fluor 555-conjugated donkey anti-sheep IgG (Invitrogen, A21436, 1/1000), HRP-conjugated anti-GFP (MACS, 130091833, 1/2000), HRP-conjugated anti-actin (Sigma, A3854, 1/10000), HRP-conjugated anti-FLAG M2 (Sigma, A8592, 1/1000), HRP-conjugated donkey anti-rabbit IgG (GE Healthcare, NA934V, 1/5000), HRP-conjugated sheep anti-mouse IgG (GE Healthcare, NXA931, 1/5000), HRP-conjugated donkey anti-guinea pig IgG (Jackson Immuno Research, 706-035-148, 1/5000) and HRP-conjugated sheep anti-mouse IgG (GE Healthcare, NXA931, , 1/5000).
Validation	Antibodies were chosen for specific applications based on suppliers' recommendations indicated on their websites and all showed bands of expected molecular weight.<br>Antibodies to ATG9A, ATG2A, ATG2B, ATG7 were validated in knock-out cell lines by immunoblotting and immunostaining.<br>The anti-TOMM20 antibody was shown by Abcam and other research publications (for example doi: 10.1038/s41467-021-22113-3) to co-localize with other mitochondrial markers in human immunostained cells.

The anti-TGN46 antibody was shown by Biorad and other research publications (for example doi: 10.3390/ijms22094936) to co-localize with other Golgi markers in human immunostained cells.
The anti-PLIN5 antibody was shown by Progen and other research publications (for example doi: 10.1038/ncomms12723) to detect PLIN5 at the expected molecular weight by immunoblotting.
The anti-PLIN3 antibody was shown by Proteintech and by us to stain lipid droplets by immunostaining and to colocalize with BODIPY 493 (probe staining neutral lipids contained inside lipid droplets). Proteintech performed the immunostaining after treating cells with oleate and visualized an increase of the lipid droplet pool (reproduced in this paper).
The anti-LC3B antibody was validated in LC3B knock-out cell lines by immunoblotting and immunostaining by Cell Signaling and by us in cell lines knocked-out for other autophagy proteins. In addition, the detection of the LC3B-II form of LC3B was significantly increased after a starvation and Bafilomycin A treatments by immunostaining and immunoblotting.
The anti-Actin antibody was shown by Sigma to detect beta-Actin at the expected molecular weight by immunoblotting and the characteristic Actin filaments by immunohistochemistry.
The anti-Flag antibody was shown by Sigma and by us to detect Flag-tagged over-expressed proteins by immunoblotting at expected molecular weight.
The anti-GFP antibody was shown by MACS and by us to detect GFP-tagged over-expressed proteins by immunoblotting at expected molecular weight.

Eukaryotic cell lines

Policy information about cell lines

Cell line source(s)	We purchased cel lines from ATCC: HeLa (CCL-2), U-2 OS (HTB-96), HEK-293 (CRL-1573).
Authentication	Manufacturers' authenticated cell lines. In addition, we assessed the cell morphology by microscopy. Aliquots of each cell line were prepared at low passage and kept frozen. Once thawed, cells were kept in culture for no more than 20 passages.
Mycoplasma contamination	We did not perform testing for mycoplasma but routinely stained cell lines with DAPI to ensure there was no bacterial or mycoplasma contamination.
Commonly misidentified lines (See ICLAC register)	We did not use misidentified cell lines.

Animals and other organisms

Policy information about studies involving animals; ARRIVE guidelines recommended for reporting animal research

Laboratory animals	Species: Caenorhabditis elegans<br>Strains: Bristol N2<br>Sex: Hermaphrodite and male<br>The C. elegans stage is 24 hours post mid-L4.
Wild animals	Study did not involve wild animals.
Field-collected samples	Study did not involve samples collected from the field.
Ethics oversight	Caenorhabditis elegans is a free-living, non-parasitic, non-pathogenic, non-infectious soil nematode animal. No ethical approval or guidance was required for C. elegans.

Note that full information on the approval of the study protocol must also be provided in the manuscript.

Flow Cytometry

Plots

Confirm that:

- [x] The axis labels state the marker and fluorochrome used (e.g. CD4-FITC).
- [x] The axis scales are clearly visible. Include numbers along axes only for bottom left plot of group (a 'group' is an analysis of identical markers).
- [x] All plots are contour plots with outliers or pseudocolor plots.
- [x] A numerical value for number of cells or percentage (with statistics) is provided.

Methodology

Sample preparation	Cells were washed, incubated with BODIPY 493 (stock solution in DMSO, used at 2 µg/ml in CM) for 25 min at 37°C, washed, trypsinized and resuspended in 1X PBS supplemented with 5 mM EDTA. Cell suspensions were strained through a 35µm, nylon strainer cap into polystyrene test tubes and kept on ice prior to analysis.

Instrument	FACS analysis was performed on an LSRFortessa flow cytometer (BD Biosciences).
Software	Fluorescence intensities were analyzed using FlowJo software.
Cell population abundance	All flow cytometry analyses were performed on 100,000 cells.
Gating strategy	Viable cells were first gated on a plot of FSC-A versus SSC-A. A gate was set based on BODIPY 493- unlabeled HeLa cells.

☒ Tick this box to confirm that a figure exemplifying the gating strategy is provided in the Supplementary Information.
